# Giant parathyroid adenoma: a case report and review of the literature

**DOI:** 10.1186/s13256-019-2257-7

**Published:** 2019-11-14

**Authors:** Mohamed S. Al-Hassan, Menatalla Mekhaimar, Walid El Ansari, Adham Darweesh, Abdelrahman Abdelaal

**Affiliations:** 1 Department of General Surgery, Hamad General Hospital, Hamad Medical Corporation, Doha, Qatar; 2 Weill Cornell Medicine-Qatar, Doha, Qatar; 3 Department of Surgery, Hamad General Hospital, Hamad Medical Corporation, Doha, Qatar; 4 College of Medicine, Qatar University, Doha, Qatar; 5 School of Health and Education, University of Skövde, Skövde, Sweden; 6 Department of Medical Imaging, Hamad General Hospital, Hamad Medical Corporation, Doha, Qatar

**Keywords:** Giant parathyroid adenoma, Parathyroidectomy, Primary hyperparathyroidism, Minimal invasive parathyroidectomy, Atypical parathyroid adenoma

## Abstract

**Background:**

Giant parathyroid adenoma is a rare type of parathyroid adenoma defined as weighing > 3.5 g. They present as primary hyperparathyroidism but with more elevated laboratory findings and more severe clinical presentations due to the larger tissue mass. This is the first reported case of giant parathyroid adenoma from the Middle East.

**Case presentation:**

A 52-year-old Indian woman presented with a palpable right-sided neck mass and generalized fatigue. Investigations revealed hypercalcemia with elevated parathyroid hormone and an asymptomatic kidney stone. Ultrasound showed a complex nodule with solid and cystic components, and Sestamibi nuclear scan confirmed a giant parathyroid adenoma. Focused surgical neck exploration was done and a giant parathyroid adenoma weighing 7.7 gm was excised.

**Conclusions:**

Giant parathyroid adenoma is a rare cause of primary hyperparathyroidism and usually presents symptomatically with high calcium and parathyroid hormone levels. Giant parathyroid adenoma is diagnosed by imaging and laboratory studies. Management is typically surgical, aiming at complete resection. Patients usually recover with no long-term complications or recurrence.

## Background

The normal parathyroid gland weighs approximately 50–70 mg. Parathyroid adenomas (PTAs) are usually small, measuring < 2 cm and weighing < 1 gm [1]. Giant PTAs (GPTAs), although rare, are most commonly defined as weighing > 3.5 gm, with some reports describing weights up to 110 gm [2, 3]. Both PTA and GPTA present with the syndrome of primary hyperparathyroidism (PHPT), the third most common endocrine disorder [4]. The pathophysiology of PHPT is autologous secretion of parathyroid hormone (PTH) by one or more of the parathyroid glands [4]. Although PHPT can be caused by parathyroid hyperplasia or carcinoma, however, around 85% of cases of PHPT are due to PTAs, and the majority of these are because of solitary PTAs, of which GPTA comprise a small number [5].

To the best of our knowledge, this case report describes the first case in the Middle East of a patient with non-ectopic GPTA presenting with visible neck swelling. This case report also reviews the published literature to report on the clinical characteristics and typical presentation of GPTA as well as diagnosis and treatment.

## Case presentation

A 52-year-old Indian woman was referred to our Surgical Endocrinology clinic at Hamad General Hospital in Doha, Qatar. She complained of a neck swelling and generalized fatigue. Laboratory results showed hypercalcemia and elevated PTH. Her past social, environmental, family, and employment history (housewife) were unremarkable. She did not smoke tobacco and never consumed alcohol. There was no past history of symptomatic kidney stones; however, a recent computed tomography (CT) scan of her abdomen and pelvis showed a 2 mm non-obstructing calculus in the lower pole calyx of her right kidney with no hydroureteronephrosis. Her past medical history indicated that she had dyslipidemia, controlled with medication; however, she was not on any other medication. On physical examination, a right-sided neck swelling was obvious on inspection; on palpation a mobile non-tender nodule could be felt, approximately 3 cm in size. The rest of the physical examination was unremarkable. A neurological examination was unremarkable. On admission, her pulse, blood pressure and temperature were normal.

Serology laboratory tests showed corrected calcium of 3.12 mmol/L, an intact PTH of 503 ng/L, vitamin D of 19.97 nmol/L, and normal thyroid-stimulating hormone (TSH) level. Her renal functions were within normal limits, serum creatinine was 67 μmol/L, and 24-hour urine calcium was 4.30 mmol/L per 24 hours. Her complete blood count (CBC) and liver laboratory findings were within normal limits. Microbiology laboratory tests were not deemed necessary.

**Fig. 1:**
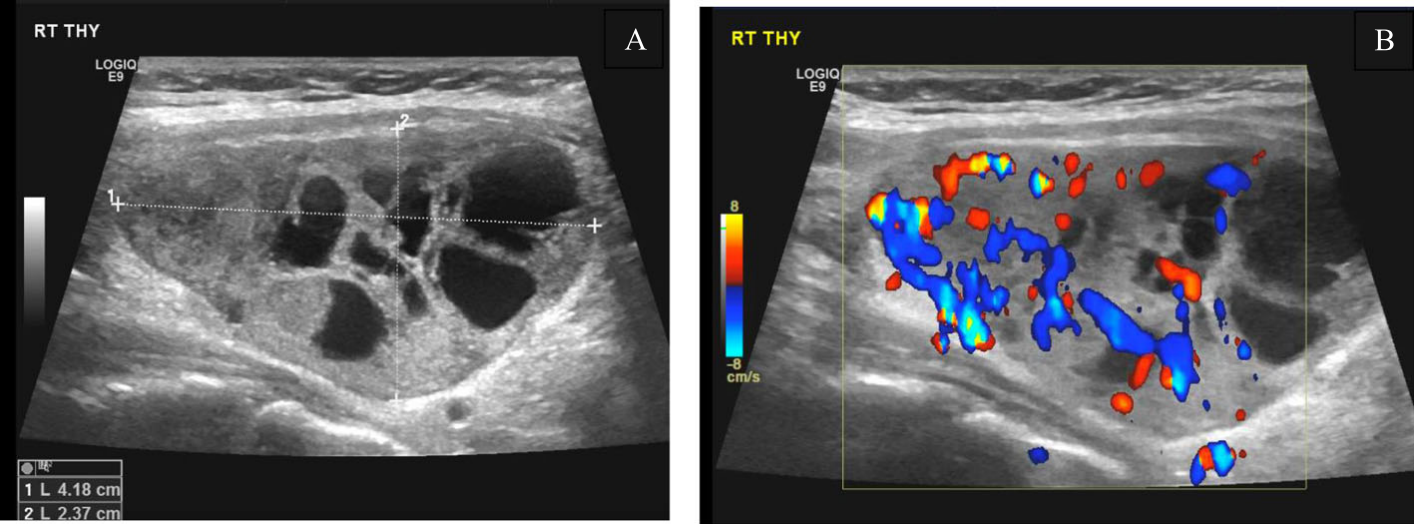
Ultrasound of the neck showing complex nodule of the right lobe with solid and cystic components (**a**) and vascularity (**b**)

**Fig. 2:**
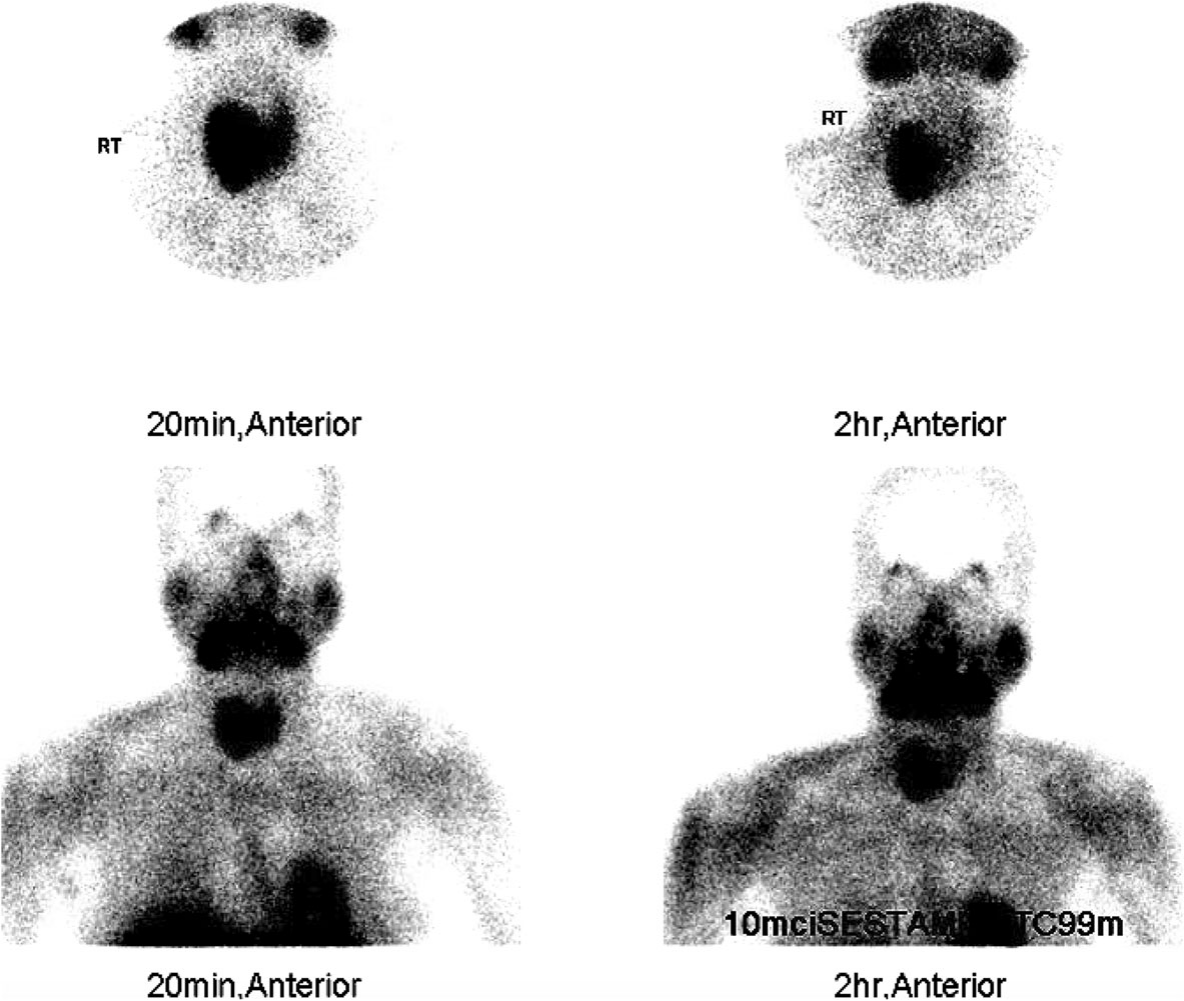
Early and late ^99m^Tc-sestamibi scintigraphy parathyroid scan images of neck and mediastinum anteriorly at 20 minutes and 2 hours showing increased focal uptake suggestive of right giant parathyroid adenoma

Imaging investigations included an ultrasound of her neck that showed a complex nodule (4.1 × 2.3 cm) with solid and cystic components, and vascularity was observed in the mid to lower pole of her right thyroid gland (Fig. 1). A parathyroid Sestamibi scan revealed tracer concentration in the thyroid tissues with more intense focal uptake observed related to the lateral side of the right thyroid lobe (Fig. 2). A delayed scan revealed residual persistent uptake, corresponding to the initially described increased focal uptake seen on the early images. These Sestamibi findings were highly suggestive of a PTA. Ultrasound-guided fine-needle aspiration (FNA) was done but was non-diagnostic.

## Surgical technique

**Fig. 3:**
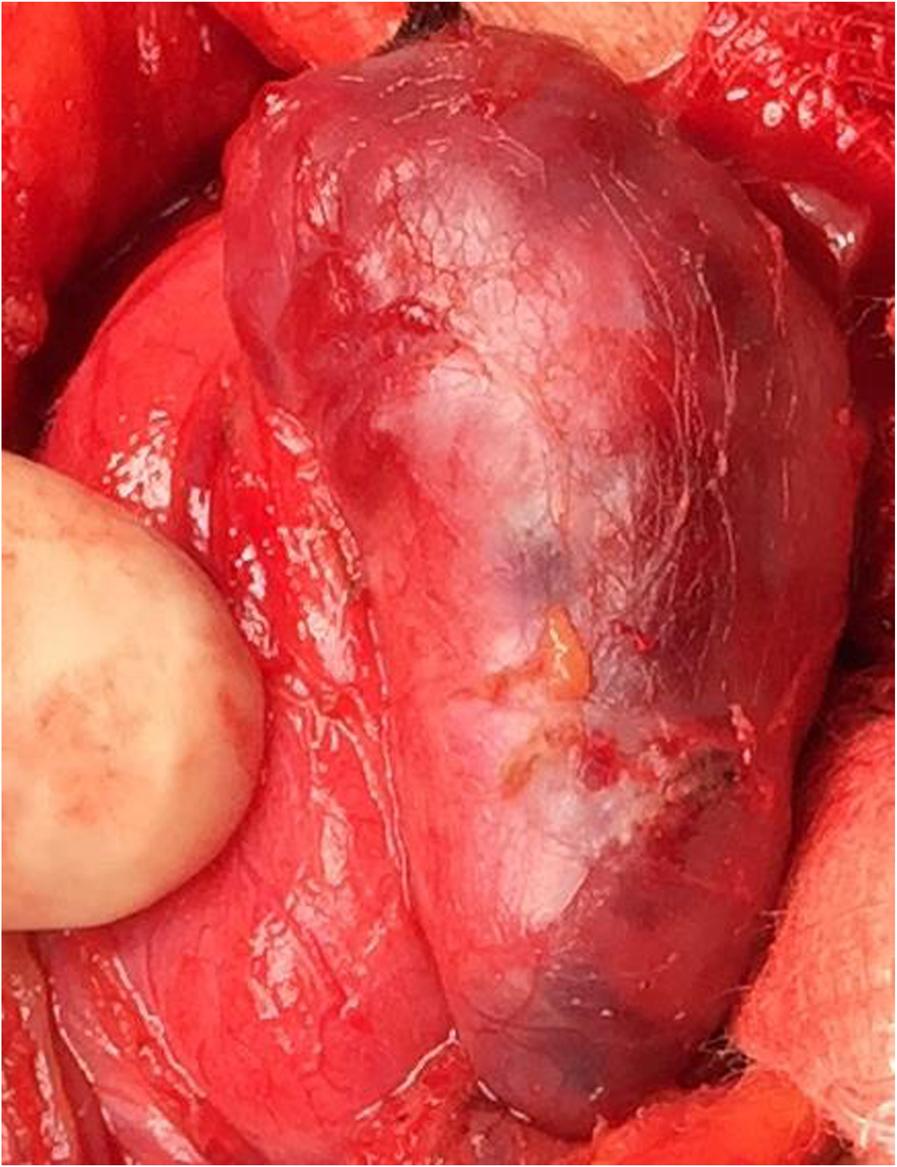
Giant parathyroid adenoma identified intraoperatively

**Fig. 4:**
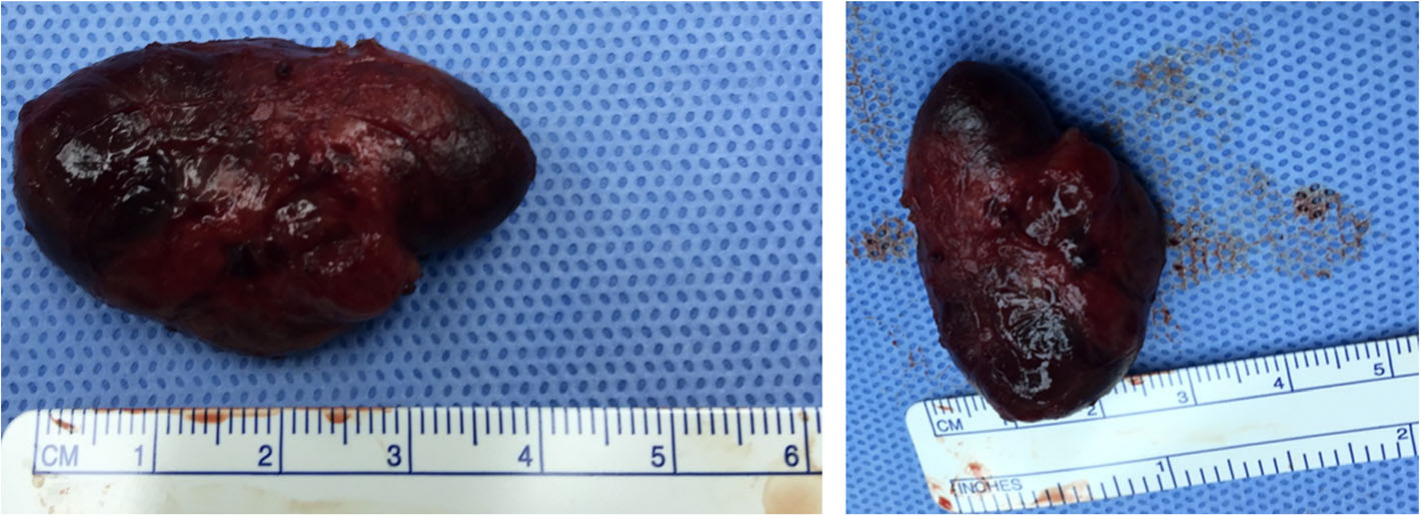
Excised giant parathyroid adenoma (4 × 2.5 × 1.5 cm)

This patient fitted two of the criteria for surgical management of PTA: (1) she had renal involvement, and, (2) serum calcium was 3.12 mmol/L (> 0.25 mmol/L from the upper limit of normal) [6]. She underwent minimally invasive parathyroidectomy (MIP) with focused exploration and excision of the right PTA under general anesthesia. A transverse collar incision was made, the surgery proceeded and the adenoma was identified and excised (Fig. 3). Intraoperative PTH (ioPTH) monitoring confirmed the excision of the adenoma as the PTH dropped from the initial pre-excision level of 546 ng/L to 239 ng/L 10 minutes after the excision, and then to 161 ng/L 20 minutes after excision, a 70% drop. Intraoperative frozen sections were sent to the pathology laboratory that confirmed PTA. Surgery was concluded; our patient recovered without any complications and was discharged on the second postoperative day. Final histopathology of the gland reported a nodule (4 × 2.5 × 1.5 cm) weighing 7.7 gm with histologic features consistent with a PTA (Fig. 4). Our patient was followed for a total of 3 years postoperatively and she remained asymptomatic and normocalcemic, without recurrence.

## Discussion

A 52-year-old woman presented with a visible palpable right-sided neck mass and generalized fatigue. Ultrasound showed a complex nodule with solid and cystic components, and a Sestamibi nuclear scan confirmed a GPTA. Focused surgical neck exploration was done and a GPTA weighing 7.7 gm was excised. This case of non-ectopic GPTA is unusual in that it presented mainly with visible palpable right-sided neck mass, which was large to the extent that the initial ultrasound suggested that it could be a thyroid nodule.

PTAs are well-reported tumors that cause PHPT. However, when their weight exceeds 3.5 gm, they are classified as GPTA [2]. Ours weighed 7.7 gm, and is considered a smaller GPTA in relation to other reported cases [7–9].

In terms of presentation, the classic presentation of PTA is with PHPT accompanied by recurrent kidney stones, and psychiatric, bone, and gastrointestinal symptoms [4]. However, this full constellation of symptoms is rarely seen nowadays due to the more frequent routine assessments of blood chemistries of patients presenting to hospital and clinics. Hence, such early detection has led to the majority of patients with PHPT now being identified early in the asymptomatic stage [4, 10]. However, our review of cases of GPTA published during the last 10 years (Table 1, 20 case reports, 22 patients with GPTA) shows that only two out of the 22 published cases were completely free of signs and symptoms of hypercalcemia [15, 17]. On the contrary, Table 1 suggests that most GPTAs presented symptomatically, ranging from vague bone/abdominal pain [5, 11, 18, 24] to more severe presentations, for example chronic depression, recurrent symptomatic kidney stones, and severe gastrointestinal symptoms [1, 9, 13, 14, 19–22]. Very rare presentations included hyperparathyroid crisis and acute pancreatitis [1, 10, 16]. Our current patient presented with neck swelling and generalized fatigue, and she had a silent kidney stone that was identified by abdominal CT, suggesting that GPTA generally presents symptomatically or with signs of hypercalcemia. This is probably related to the higher levels of calcium produced by the larger tumor mass.

**Table 1:** Literature review: Case studies of giant parathyroid adenoma (2009–2019)

Study^*^	Sex	Age, years	Side	Presentation	Ca (mmol/L)/PTH (ng/L)	Radiology	Treatment	IPTH	Dimensions (mm)	Weight (g)	Pathology	Postoperative complications^*a*^
Thyroidal												
Aggarwal *et al*., 2009 [11] India	F	33	L	Visible swelling, palpable nodule, bone pain, R humerus and R pelvic fractures	2.65/762	US: well-defined hypoechoic lesion, posterior to left lobe thyroid	Parathyroidectomy (not specified)	—	95 × 50 × 35	102	Chief cell adenoma	Symptomatic hypocalcemia
Salehian *et al*., 2009 [12] Iran	F	53	R	Visible swelling, bone pain, nausea, vomiting, weight loss	3.65/1624	US neck: heteroechoic mass, inferior right lobe (2 × 4.8 × 3 cm); ^99m^Tc-MIBI: abnormal collection of tracer in R side of neck	Neck exploration and parathyroidectomy (collar neck incision)	—	55 × 35 × 20	30	PTA	Nil
Sisodiya *et al*., 2011 [13] India	F	52	R	Recurrent vomiting	4.25/598	US: large hypoechoic lesion in right paratracheal region with retrosternal extension	Parathyroidectomy, low anterior cervical approach	Mentioned in discussion	39 × 20 × 17	—	—	Hypocalcemia
Asghar *et al*., 2012 [14] Pakistan	F	55	L	Parathyroid crisis^*b*^ Palpable nodule	5.75/1182	US: large cyst (6 × 3.7 cm) on left side with thrombosis of IJV; MIBI: cystic lesion in left side neck displacing the thyroid gland on the right; CT: large hypodense lesion left side of neck with peripheral enhancement, retrosternal extension and mass effect with deviation of trachea and thrombosis of LIJV	Parathyroidectomy T-shaped incision 10 suspicious-looking lymph nodes also removed from levels 7 and 8 (by ENT and thoracic surgery teams)	—	110 × 70 × 60	—	PTA with prominent cystic degeneration; no lymph node metastasis	Nil
Vilallonga *et al*., 2012 [10] Spain	F	19	L	Parathyroid crisis	3.55/1207	US: 47 × 22 mm nodule in left thyroid lobe	Hemithyroidectomy (it was intrathyroidal)	Available, not used	Max. diameter 30	70	Intrathyroidal PTA	None Calcium IV d1, oral d2
Neagoe *et al*., 2014 [1] Romania (3 cases)	M/F/F	57/60/33	R/L/R	C 1: Bone pain, abdominal pain, nausea, palpable nodule C 2: Parathyroid crisis, palpable nodule C 3: Recurrent kidney stones, brown tumor of tibia	C 1: 3.54/1780 C 2: 4.04/863 C 3: 3.15/1174	MIBI: detected adenomas in the 3 cases	Bilateral neck exploration and parathyroidectomy	Not feasible^c^	C 1: 50 × 30 × 20 C 2: 55 × 40 × 30 —	C 1: 30.6 C 2: 35.2 C 3: > 30	2 PTA; 1 partially cystic PTA	C 1: Hungry bones syndrome C 2: Mild hypocalcemia and hungry bones syndrome C 3: Mild hypocalcemia
Haldar *et al*., 2014 [15] UK	F	61	L	Asymptomatic	3.17/179.2	US: 6 cm mass in L inferior cervical location; MIBI: persistent activity in same location; SPECT: tubular structure in superior mediastinum	Parathyroidectomy (selective) 4 cm left collar neck incision	—	65 × 30 × 15	12	PTA	Nil
Garas *et al*., 2015 [5] UK	F	53	L	Bone pain, palpable nodule	3.98/4038	US: lobular well-defined hypoechoic lesion behind L lower pole of thyroid gland; MRI: left inferior PTA, extends deep into mediastinum	Parathyroidectomy (transverse cervical incision)	Done – 94% reduction in 25 minutes	Max. diameter 70	27	Chief cell PTA	Nil
Rutledge *et al*., 2016 [7] Ireland	F	21	R	Enlarging neck mass, constipation, palpable nodule	2.73/1305.1	MIBI: lesion posterior to right lobe of thyroid with concentrated tracer	R thyroid lobectomy and parathyroidectomy with level 6 neck dissection (suspected carcinoma)	—	80 × 55 × 30	58.8	Atypical PTA	Symptomatic hypocalcemia, hungry bone syndrome
Krishnamurthy *et al*., 2016 [16] India	M	50	L	Recurrent attacks of acute pancreatitis, palpable fullness	2.77/669	CT: 6 × 4 cm mass in L paratracheal region with extension to superior mediastinum; PET–CT: isolated uptake, left paratracheal region; MIBI: localized to L inferior parathyroid gland; Preoperative FNA-C was done^*d*^	Parathyroidectomy via transcervical approach	—	Max. diameter 60	20	PTA	Hypocalcemia
Castro *et al*., 2017 [17] Spain	F	40	L	Asymptomatic, palpable nodule	3.35/825	US: solid lesion behind L thyroid lobe; SPECT: intense uptake, back of L thyroid lobe in early and late phases	Parathyroidectomy (not specified)	Done, 90% reduction	64 × 16 × 20	10.8	PTA	Hypocalcemia
Sahsamanis *et al*., 2017 [18] Greece	F	42	L	Abdominal pain	2.60/151	US: enlarged parathyroid gland on lower side of cervical region; MIBI: large concentrations of radiotracer in the same location	Minimally invasive parathyroidectomy	Not done	33 × 20 × 14	5.39	PTA	Nil
Mantzoros *et al*., 2018 [19] Greece	F	73	R	Neck swelling, bone pain	3.63/1629	US: hypoechoic nodule at inferior pole of the right thyroid; MIBI: hyper functioning rightlower parathyroid gland	Minimally invasive parathyroidectomy	Done, 95% reduction 20 minutes after removal	50 × 25 × 25	30	PTA	Hungry bone syndrome
Mediastinal												
Migliore *et al*., 2013 [8] Italy	F	65	R	Persistent hypercalcemia^*e*^	Both elevated	CT: 7 cm mass in posterior mediastinum; MIBI: confirmed the CT finding	Video-assisted minithoracotomy	—	—	95	PTA	Nil
Taghavi Kojidi *et al*., 2016 [20] Iran	M	70	Mid	Anorexia, nausea, bone pain, constipation, symptomatic kidney stones, polydipsia	3.60/930	US: multiple isoechoic nodules, no parathyroid glands seen; MIBI: focal radiotracer accumulation, midline anterior chest wall; CT: soft tissue density mass, mild enhancement, anterior midline, xiphoid level	Surgical removal (not specified) ^*f*^	—	—	75	Active parathyroid lesion	Hypocalcemia
Pecheva *et al*., 2016 [21] UK	F	72	R	Depression, severe osteoporosis (T = −3.2)	3.02/250.8	US: no parathyroid lesion; MIBI: no evidence of PTA; CT: complex cystic solid mass in the mediastinum	Parathyroidectomy via VATS	Not used, emergency	—	19	PTA	Hoarseness, bovine cough
Talukder *et al*., 2017 [22] India	F	49	Mid	Brown tumor	14.07/1000	US: no abnormal parathyroid gland; MIBI: tracer-avid lesion in anterior mediastinum; PET-CT: ectopic parathyroid tissue in anterior mediastinum behind manubrium sterni	Parathyroidectomy via cervical collar incision and hemisternotomy	—	40 × 30 × 20	12	Neuroendocrine cell tumor	Nil
Garuna Murthee *et al*., 2018 [9] UK	M	72	Mid	Anorexia, lethargy, abdominal cramps, constipation, weight loss	15.19/1867.1	CXR: sizeable mediastinal mass; CT: 9 cm solid cystic anterior mediastinal tumor; MIBI: heterogeneous tracer uptake in the mediastinal mass	Medial sternotomy and total thymectomy	—	Maximum diameter 78	220	Intrathymic PTA	Nil
Miller *et al*., 2019 [23] UK	M	53	Mid	Asymptomatic renal stones	11.22/179.2	MIBI: linear region of increased intensity in the left mediastinum	Parathyroidectomy via transcervical excision	Done, 81% reduction after 10 minutes	80 × 30 × 30	30.9	PTA	Nil

— not reported, cannot be inferred, *C1* Case 1, *C2* Case 2, *C3* Case 3, *CT* computed tomography, *CXR* chest X-ray, *ENT* otolaryngology, *F* female, *FNA-C* fine-needle aspiration cytology, *IPTH* intraoperative parathyroid hormone, *IJV* internal jugular vein, *L* left, *M* male, *Mid* midline, *MIBI* Tc^99m^-sestamibi scintigraphy scan, *PET* positron emission tomography, *PTA* parathyroid adenoma, *PTH* fine-needle aspiration cytology, *R* right, *SPECT* single photon emission computed tomography, *US* ultrasound, *VATS* video-assisted thoracoscopic surgery

^*^ Due to space limitations, only the first author is mentioned

^*a*^ All of the cases had asymptomatic patients with normalized Ca and fine-needle aspiration cytology on follow up (except Haldar + Sisodya – Ca only)

^*b*^ Parathyroid crisis comprises anorexia, urinary frequency, severe nausea, vomiting, constipation

^*c*^ Done 1 hour postoperative for 2 cases, found to be normal

^*d*^ Preoperative fine-needle aspiration cytology showed a benign epithelial lesion that could not be further characterized

^*e*^ Patient had previous total thyroidectomy for goiter associated with hypercalcemic syndrome (exploration had showed four normal parathyroid glands)

^*f*^ Patient had previous total parathyroidectomy, thymectomy, and right hemithyroidectomy

In terms of physical examination, the majority of GPTAs in the neck had a visible and palpable mass in the neck [7, 11, 13, 14, 17, 19, 20, 24, 25]. Their large size is one of the reasons a clinician may suspect thyroid disease before reviewing the laboratory results, as palpable nodules are more common in the thyroid. In our case, a swelling was readily visible on inspection and palpable on physical examination.

As for diagnostic laboratory studies, GPTA investigations start with serum calcium and PTH and proceed to imaging for localization (Table 1). Hypercalcemia and elevated PTH are hallmarks of PHPT [4], in agreement with our review where all cases had elevated calcium and PTH laboratory values [18]. A positive correlation between the size of a PTA and preoperative PTH and calcium levels has also been reported [2, 26, 27]. Calva-Cerqueira *et al.* (2007) concluded that if preoperative PTH is > 232 ng/L, there is 95% likelihood of finding a PTA weighing > 250 mg [28]. This is valuable, as surgeons can have an idea of tumor size preoperatively. As for histopathology, although FNA cytology (FNA-C) is increasingly used in the diagnosis of parathyroid pathology [29], its limitation is that FNA-C cannot distinguish between different types of parathyroid disease [30]. Our preoperative FNA-C was non-diagnostic, similar to others where preoperative FNA-C was non-diagnostic [16].

In terms of imaging, localizing a GPTA is imperative to guide management. The most commonly used method is a combination of neck ultrasound and ^99m^Tc-sestamibi scintigraphy (MIBI) scan. The limitation of neck ultrasound in GPTA is that it may not show the extent of a lesion; when the GPTA is ectopic, a neck ultrasound will show no finding [5]. In mediastinal GPTA, neck ultrasound only rules out a neck lesion but does not otherwise aid in localization [20–22]. In neck GPTA, the combination of a MIBI scan and neck ultrasound effectively localizes the GPTA and allows for guided neck exploration [27]. Ultrasound alone predicts GPTA location with 79% accuracy; combining ultrasound, MIBI, and CT increases the accuracy of localization to 82% [2]. We agree, as in our patient, that combining neck ultrasound and MIBI scan accurately localizes the GPTA preoperatively. MIBI scans are more likely to localize GPTA in patients with higher preoperative PTH and larger GPTA size; an adenoma correctly localized by MIBI has a 95% likelihood of weighing > 5.5 gm [28]. As for the location, in agreement with most studies, our GPTA was not ectopic [2]; however, Table 1 shows that the mediastinum is a common location for ectopic GPTAs, suggesting that such GPTAs arise in the inferior gland [31, 32].

For the management, the 2014 US National Institutes of Health (NIH) guidelines for PTA management are either medical or surgical if it fulfills specific criteria [6]. As with all the cases in Table 1, our case fulfilled the symptoms and laboratory criteria for surgery. We undertook MIP, in agreement that it is the preferred procedure [19], and ioPTH monitoring, to confirm removal of the PTA before closure. Although the MIP/ioPTH combination is a gold-standard treatment, comparable outcomes of MIP with and without ioPTH monitoring have been reported [33], with some studies suggesting the benefit of ioPTH monitoring is only for patients with equivocal imaging [34]. This might be an important feature to consider when institutions seek resource utilization and cost savings. In agreement with the point that ioPTH might be beneficial only in equivocal imaging findings, ioPTH monitoring seems to have limited use in GPTA. Only in five instances was ioPTH monitoring undertaken [5, 13, 17, 19, 23] (Table 1), probably because preoperative imaging and intraoperative visualization in GPTA leave little doubt about the location [28], and because of a high likelihood of single gland disease [2]. At our institution, PTA standard intraoperative practice is ioPTH monitoring and frozen section to confirm excision.

Postoperatively, larger sized PTAs may be associated with a higher incidence of postoperative hypocalcemia [26]. Table 1 agrees with this, showing that hypocalcemia occurred in cases of larger GPTA. Hungry bone syndrome, a severe but rare form of postoperative hypocalcemia, occurred in four cases (Table 1), all of which had GPTAs weighing > 30 gm. Patients with smaller GPTAs were less likely to have postoperative hypocalcemia. Our patient presented with a 7.7 gm GPTA, considered a smaller GPTA in relation to other reported cases [7–9], which could explain why it had a less severe presentation as well as outcome after surgery compared with the other cases. Postoperatively, our patient, due to the smaller GPTA, became normocalcemic with normal PTH, and was not discharged on any calcium repletion therapy. Our patient was followed for a total of 3 years postoperatively and she remained asymptomatic and normocalcemic, without recurrence. This fits with outcomes reported in a study following patients for an average of 40 months, where all patients remained normocalcemic and there was no recurrence during this time, even in those with suspicious histologic features [27].

## Conclusion

GPTA is a rare subset of PTAs that weigh > 3.5 gm, it is benign, but can manifest with the symptoms of extreme hypercalcemia. From our literature review, we conclude that GPTA generally presents symptomatically, with high preoperative PTH and serum calcium directly proportional to the adenoma weight. The most accurate method for localizing a GPTA is a combination of neck ultrasound and MIBI scan. MIP with intraoperative PTH monitoring is the suggested management, although the need for the ioPTH monitoring is debatable in GPTA due to their large size and accuracy of preoperative imaging.

## Data Availability

Data sharing is not applicable to this article as no datasets were generated or analyzed during the current study.

## Abbreviations

CBC: Complete blood count
CT: Computed tomography
FNA: Fine-needle aspiration
FNA-C: Fine-needle aspiration cytology
GPTA: Giant parathyroid adenoma
ioPTH: Intraoperative parathyroid hormone
MIBI: ^99m^Tc-sestamibi scintigraphy
MIP: Minimally invasive parathyroidectomy
NIH: US National Institutes of Health
PHPT: Primary hyperparathyroidism
PTA: Parathyroid adenoma
PTH: Parathyroid hormone
TSH: Thyroid-stimulating hormone

## References

[1] Neagoe RM, Sala DT, Borda A, Mogoanta CA, Muhlfay G. Clinicopathologic and therapeutic aspects of giant parathyroid adenomas - three case reports and short review of the literature. Romanian J Morphol Embryol. 2014;55(2 Suppl):669–74. 25178342

[2] Spanheimer PM, Stoltze AJ, Howe JR, Sugg SL, Lal G, Weigel RJ. Do giant parathyroid adenomas represent a distinct clinical entity? Surgery. 2013;154(4):714–8; discussion 8-9 10.1016/j.surg.2013.05.013 PMC3787983 23978594

[3] Power C, Kavanagh D, Hill AD, O'Higgins N, McDermott E. Unusual presentation of a giant parathyroid adenoma: report of a case. Surg Today. 2005;35(3):235–7. 10.1007/s00595-004-2902-6 15772795

[4] Madkhali T, Alhefdhi A, Chen H, Elfenbein D. Primary hyperparathyroidism. Ulus Cerrahi Derg. 2016;32(1):58–66. 10.5152/UCD.2015.3032 PMC4771429 26985167

[5] Garas G, Poulasouchidou M, Dimoulas A, Hytiroglou P, Kita M, Zacharakis E. Radiological considerations and surgical planning in the treatment of giant parathyroid adenomas. Ann R Coll Surg Engl. 2015;97(4):e64–6. 10.1308/003588415X14181254789682 PMC4473887 26263956

[6] Bilezikian JP, Brandi ML, Eastell R, Silverberg SJ, Udelsman R, Marcocci C, *et al.* Guidelines for the management of asymptomatic primary hyperparathyroidism: summary statement from the Fourth International Workshop. J Clin Endocrinol Metab. 2014;99(10):3561–9. 10.1210/jc.2014-1413 PMC5393490 25162665

[7] Rutledge S, Harrison M, O'Connell M, O'Dwyer T, Byrne MM. Acute presentation of a giant intrathyroidal parathyroid adenoma: a case report. J Med Case Rep. 2016;10(1):286. 10.1186/s13256-016-1078-1 PMC5070099 27756436

[8] Migliore M, Pulvirenti G, Okatyeva V, Cannizzaro MA. Persistent hyperparathyroidism owing to a giant parathyroid adenoma in posterior mediastinum. Surgery. 2013;154(1):132–3. 10.1016/j.surg.2012.04.003 22698932

[9] Garuna Murthee K, Tay WL, Soo KL, Swee DS. A Migratory Mishap: Giant Mediastinal Parathyroid Adenoma. Am J Med. 2018;131(5):512–6. 10.1016/j.amjmed.2018.01.003 29360437

[10] Vilallonga R, Zafon C, Migone R, Baena JA. Giant intrathyroidal parathyroid adenoma. J Emerg Trauma Shock. 2012;5(2):196–8. 10.4103/0974-2700.96497 PMC3391849 22787355

[11] Aggarwal V, Mishra A, Bhargav PR, Ramakant P. Giant parathyroid adenoma. ANZ J Surg. 2009;79(1–2):91. 10.1111/j.1445-2197.2008.04815.x 19183396

[12] Salehian M, Namdari O, Mohammadi SS, Feazli YH. Primary hyperparathyroidism due to a giant parathyroid adenoma: a case report. Int J Endocrinol Metabol. 2009;9(2):101–5.

[13] Sisodiya R, Kumar S, Palankar N, BVD. Case report on giant parathyroid adenoma with review of literature. Indian J Surg. 2011;75(Suppl 1):21–2. 10.1007/s12262-011-0306-6 PMC3693292 24426500

[14] Asghar A, Ikram M, Islam N. A case report: Giant cystic parathyroid adenoma presenting with parathyroid crisis after Vitamin D replacement. BMC Endocr Disord. 2012;12:14. 10.1186/1472-6823-12-14 PMC3462668 22840059

[15] Haldar A, Thapar A, Khan S, Jenkins S. Day-case minimally invasive excision of a giant mediastinal parathyroid adenoma. Ann R Coll Surg Engl. 2014;96(5):e21–3. 10.1308/003588414X13946184900480 PMC4473961 24992407

[16] Krishnamurthy A, Raghunandan GC, Ramshankar V. A rare case of giant parathyroid adenoma presenting with recurrent episodes of pancreatitis. Indian J Nucl Med. 2016;31:36–8. 10.4103/0972-3919.172355 PMC4746839 26917892

[17] Castro MA, López AA, Fragueiro LM, García NP. Giant parathyroid adenoma: differential aspects compared to parathyroid carcinoma. Endocrinol Diabetes Metab Case Rep. 2017;2017:1. 10.1530/EDM-17-0041 PMC5420060 28491324

[18] Sahsamanis G, Gkouzis K, Samaras S, Pinialidis D, Dimitrakopoulos G. Surgical management of a giant parathyroid adenoma through minimal invasive parathyroidectomy. A case report. Int J Surg Case Rep. 2017;31:262–5. 10.1016/j.ijscr.2017.01.064 PMC5310179 28199935

[19] Mantzoros I, Kyriakidou D, Galanos-Demiris K, Chatzakis C, Parpoudi S, Sapidis N, *et al.* A Rare Case of Primary Hyperparathyroidism Caused by a Giant Solitary Parathyroid Adenoma. Am J Case Rep. 2018;19:1334–7. 10.12659/AJCR.911452 PMC6238585 30405093

[20] Taghavi Kojidi H, Vagharimehr N, Mohseni S, Pajouhi M, Mohajeri-Tehrani MR. Unusual Ectopic Parathyroid Adenoma: A Case Report. Acta Med Iran. 2016;54(8):547–50. 27701727

[21] Pecheva M, Mahendran K, Kadlec J, Lofthouse M, Van Tornout F. Mediastinal giant parathyroid adenoma-a minimally invasive mediastinal surgical approach for an emergency presentation. Ann Cardiothorac Surg. 2016;5(1):70–3. 10.3978/j.issn.2225-319X.2015.08.12 PMC4740097 26904438

[22] Talukder S, Behera A, Bhadada SK, Mitra S. Giant mediastinal parathyroid adenoma presenting as bilateral brown tumour of mandible: a rare presentation of primary hyperparathyroidism. BMJ Case Rep. 2017;2017(11):bcr–2017. 10.1136/bcr-2017-220722 PMC5665274 29066636

[23] Miller BJ, Isaacs K, Khan E, Palazzo FF. Transcervical excision of a giant mediastinal parathyroid adenoma. BMJ Case Rep. 2019;12(2) 10.1136/bcr-2018-228292. PMC6381945 30737330

[24] Korukluoglu B, Ergul E, Yalcin S. Giant intrathyroidal parathyroid cystic adenoma. J Pak Med Assoc. 2008;58(10):592. 18998321

[25] Desigan S, Syed R, Conway GS, Kurzawinski TR, Bomanji JB. Giant cervical parathyroid adenoma mimicking a sternocleidomastoid mass and presenting as a brown tumor of the mandible. Clin Nucl Med. 2007;32(4):306–8. 10.1097/01.rlu.0000257293.00744.3c 17413582

[26] Zamboni WA, Folse R. Adenoma weight: a predictor of transient hypocalcemia after parathyroidectomy. Am J Surg. 1986;152(6):611–5. 10.1016/0002-9610(86)90436-8 3789284

[27] Abdel-Aziz TE, Gleeson F, Sadler G, Mihai R. Dwarfs and Giants of Parathyroid Adenomas-No Difference in Outcome After Parathyroidectomy. J Surg Res. 2019;237:56–60. 10.1016/j.jss.2018.12.021 30694792

[28] Calva-Cerqueira D, Smith BJ, Hostetler ML, Lal G, Menda Y, O'Dorisio TM, *et al.* Minimally invasive parathyroidectomy and preoperative MIBI scans: correlation of gland weight and preoperative PTH. J Am Coll Surg. 2007;205(4 Suppl):S38–44. 10.1016/j.jamcollsurg.2007.06.322 17916517

[29] Heo I, Park S, Jung CW, Koh JS, Lee SS, Seol H, *et al.* Fine needle aspiration cytology of parathyroid lesions. Korean J Pathol. 2013;47(5):466–71. 10.4132/KoreanJPathol.2013.47.5.466 PMC3830994 24255635

[30] Kumari N, Mishra D, Pradhan R, Agarwal A, Krishnani N. Utility of fine-needle aspiration cytology in the identification of parathyroid lesions. J Cytol. 2016;33(1):17–21. 10.4103/0970-9371.175490 PMC4782397 27011436

[31] Phitayakorn R, McHenry CR. Incidence and location of ectopic abnormal parathyroid glands. Am J Surg. 2006;191(3):418–23. 10.1016/j.amjsurg.2005.10.049 16490559

[32] LoPinto M, Rubio GA, Khan ZF, Vaghaiwalla TM, Farra JC, Lew JI. Location of abnormal parathyroid glands: lessons from 810 parathyroidectomies. J Surg Res. 2017;207:22–6. 10.1016/j.jss.2016.08.045 27979480

[33] Mihai R, Palazzo FF, Gleeson FV, Sadler GP. Minimally invasive parathyroidectomy without intraoperative parathyroid hormone monitoring in patients with primary hyperparathyroidism. Br J Surg. 2007;94(1):42–7. 10.1002/bjs.5574 17083106

[34] Khan AA, Khatun Y, Walker A, Jimeno J, Hubbard JG. Role of intraoperative PTH monitoring and surgical approach in primary hyperparathyroidism. Ann Med Surg (Lond). 2015;4(3):301–5. 10.1016/j.amsu.2015.08.007 PMC4556812 26468374

